# Unrecorded Butterfly Species and Potential Local Extinctions: The Role of Citizen Science and Sampling

**DOI:** 10.1002/ece3.71023

**Published:** 2025-02-17

**Authors:** S. Alberti, A. Pollo, C. Cerrato, R. Viterbi, E. Balletto, L. Dapporto, S. Bonelli, I. Piccini

**Affiliations:** ^1^ Department of Life Sciences and Systems Biology University of Turin Turin Italy; ^2^ Gran Paradiso National Park Turin Italy; ^3^ Department of Biology University of Florence Florence Italy; ^4^ Department of Zoology Poznań University of Life Sciences Poznań Poland

**Keywords:** altitude, citizen science, conservation, ecological traits; historical data, parks, temporal distribution

## Abstract

Estimating species extinction risk is crucial to reverse biodiversity loss and to adopt proper conservation measures. Different sources may play a pivotal role in prioritising species conservation. Recently, citizen science demonstrated a substantial role, especially when it comes to butterflies. This study examines species records and richness in Aosta Valley, which represents one of the highest mountain areas in Europe. Through 30,351 data points from 1825 to 2022, the impact and efficiency of three groups of data sources were investigated: literature (i.e., publications and collections), sampling (butterfly experts' recording), and citizen science (open‐source databases). The study also aims to assess the extinction potential of the butterflies in relation to functional traits. The results showed that even if there were significant differences in the number of records between the three sources, there were no significant differences for species recorded. Moreover, 2.9% of the butterfly community risks extinction, and it is related to some response traits. Indeed, extinction risks increase when the altitudinal range decreases and for multivoltines. In conclusion, citizen science has a strong impact on the amount of data and could be exploited to fill data gaps at low/medium altitudes. However, professional sampling is needed to focus on species no longer reported, and in particular on species that are difficult to identify, have specific distributions or particular traits (e.g., limited altitudinal range). Using different data sources, extinction risk estimation, and trait analysis, it is possible to prioritise studies on some species using different efforts (sampling and/or citizen sciences).

## Graphical abstract

30,351 data from 1825 to 2022 were used to assess community changes in Aosta Valley. 2.9% of the butterfly community risks extinction. Butterfly extinction risks increase when the altitudinal range decreases.

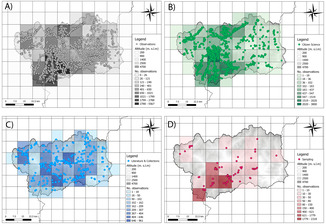

## 1 Introduction

Biodiversity is undergoing a rapid decline due to human activities such as habitat loss, invasive species, climate change, and overexploitation of resources (Tilman et al. 2017). Insects, including butterflies, are experiencing worldwide reductions in abundance and local species richness (Thomas et al. 2004; Forister et al. 2011; Habel et al. 2016). Several studies in Central and Northern Europe have recorded a general trend of butterfly decline and simplification of communities (Ulrich et al. 2024; Habel et al. 2022, 2019; Thomas 2016). Few studies have investigated the phenomenon in the Mediterranean region (but see Melero et al. 2016) and in the Alps (but see Bonelli et al. 2022 and Habel et al. 2023). Butterfly community changes and local extinctions in the highest mountain areas of Europe are still less studied.

Species respond to environmental changes in three possible ways: (i) by acclimating to new conditions; (ii) by shifting their distributions towards suitable sites; (iii) by shifting their phenology (Kremen et al. 2007; Settele et al. 2016; Suzuki‐Ohno et al. 2020). If they fail to respond to changes, declines occur that can ultimately lead to local extinctions (Bonelli et al. 2011). Morphological, physiological, phenological, or behavioral features of species can influence their responses to environmental changes (Díaz et al. 2013; Piccini et al. 2018). Butterfly extinction risks have been predicted and evaluated in different ways: through their distribution (Franzén and Johannesson 2007), their functional traits (Palash et al. 2022; Franke et al. 2022), population sizes (McLaughlin et al. 2002), genetic analysis (Nieminen et al. 2001; Piccini, Pellegrino, et al. 2024; Piccini, Pollo, et al. 2024) and on first and last species recordings (Theng et al. 2020 based on Chisholm et al. 2016). The last method relies on large datasets that include historical data and, for this reason, is not so common but present (see also van Tongeren et al. 2023). The likelihood that a species is truly extinct is higher if the species is not recorded for several years. To identify declines and changes in species distribution and ideally identify the drivers of such changes, different sources of data can be conveniently gathered. Among them, citizen science data can play a crucial role in obtaining large quantities of data (Pocock et al. 2017). Biodiversity studies focusing on fauna and flora have already successfully used different sources of data to estimate decline and extinctions (Theobald et al. 2015; Fontaine et al. 2022). In particular, citizen science has been shown to work particularly well in collecting presence data on some animal taxa such as birds and butterflies (Ries and Oberhauser 2015; Prudic et al. 2017; Adler et al. 2020; Labadessa et al. 2021; Sanderson et al. 2021). Some citizen science projects can provide data on species abundance, in addition to distribution, using standardized methods (e.g., Kaartinen et al. 2013).

Butterflies (Lepidoptera) have been studied since the 18th century; in fact, they are among the most frequently monitored groups along with birds and vascular plants in Europe (Moussy et al. 2022; Thomas 2005). In addition to this, some butterflies might be considered umbrella species for other insect taxa (Piccini, Pittarello, Di Pietro, et al. 2022) and as bio‐indicators due to their distribution in a wide variety of habitats and their sensitivity to environmental variations (Thomas 1994; Swaay et al. 1999; Hambler et al. 2004; van Swaay et al. 2006; Devictor et al. 2012; Macrì et al. 2023; Bruschini et al. 2024). The main drivers of butterfly decline are habitat loss and degradation, chemical pollution, and climate change (Warren et al. 2021; Piccini, Pittarello, Gili, et al. 2022). Species characterised by some trait values related to their life histories (e.g., low dispersal, large body size, short flight period, univoltine, feeding specialists) are particularly sensitive to changes in climatic and land‐use changes, making them more prone to extinction (Carnicer et al. 2013; Aguirre‐Gutiérrez et al. 2016; Bruschini et al. 2024).

This is one of the first studies that investigates butterfly communities and local extinction in the highest mountain areas of Europe, i.e., Autonomous Region of Aosta Valley (NW of Italy). Specifically, this study aims to evaluate (i) the changes in spatial and temporal distribution (1825–2022) of butterfly data (record abundance and species richness) according to the data sources; (ii) how data sources and functional traits affect record abundance and species extinction risk; (iii) overall how the number of records and species extinction risk is affected by functional traits; and (iv) the potential of local extinction for the entire community in the study area.

## 2 Materials and Methods

### 2.1 Study Area

The Autonomous Region of Aosta Valley includes 20% of mountainous territory over 1500 m a.s.l., and forests occupy about 30% of the total area (Camerano et al. 2007). The topography, mostly the surrounding mountains, does not allow humid air to pass through, resulting in a more arid climate in the central area. During winter, at the highest altitudes, precipitation is mainly snowy with temperatures between −5°C and 6°C, while in summer there are frequent thunderstorms and temperatures rise to between 13°C and 28°C (Mercalli et al. 2003).

### 2.2 Data Collection

Records of all butterfly species (Lepidoptera: Papilionoidea) recorded for the Aosta Valley were collected by retrieving data from faunistic literature, collections, geographically referenced open‐source databases (e.g., iNaturalist and GBIF) and sampling coordinated by the authors. The regional dataset presents species names following the Italian nomenclature (Balletto et al. 2014; Dapporto et al. 2022), the year of sampling (1825–2022, 118 years in total), the source of data, and the coordinates in decimal degrees, precise whenever possible or deduced from the description when available. We added wingspan, voltinism, and the number of host plant genera for each species as functional traits and altitudinal ranges (Paolucci 2013; Middleton‐Welling et al. 2020). All maps, data extraction, and cartographic operations were carried out with QGIS (v. 3.16.7).

We used three source categories:

- *Literature & Collections*, semi‐structured and qualitative data, without constraints on time or location. Some experience in butterfly identification is typically required, as the goal is usually to document all species present within a broad area or to pinpoint new locations for rare species. This source includes data from Checklist and distribution of the Italian fauna (CKmap), which contains faunistic literature data and records of butterfly specimens held in major national collections. The CKmap database was published in 2007 with 60,000 records (Balletto et al. 2007) and it is continuously updated by EB and SB. As of December 2021, it contained 335,499 occurrence data at a scale of ~10 × 10 km^2^, with coordinates representing the centroid of the Universal Transverse Mercator (UTM) grid. Moreover, we updated the dataset, including new validated records and recent publications. We assigned the year of publication for 2422 records in our database from literature or collections that did not show the sampling year. These observations were categorized as “Year assigned” We removed from analysis 238 data for which it was not possible to deduce the year of record (e.g., collections and faunistic literature).
- *Citizen Science*, unstructured data, and entirely opportunistic, with no restrictions on time or location and no specific objectives. This source includes all data till November 2022 from open‐source databases (iNaturalist and Observation.org), Butterfly Monitoring Scheme Italy (hereafter BMS Italy; a single transect, included experimentally due to its recent introduction) and Park Rangers. iNaturalist is a citizen science portal that allows data (usually pictures, location and date) to be uploaded by registered users, both with precisely shared and obscured coordinates (with a buffer of 27 km). We personally validated all data from iNaturalist that present at least one image. On the other hand, Observation.org is a portal with precise coordinates where data validation is done by experts before the upload, and we download them using the GBIF platform (other data from GBIF were not used because they could not be validated and not directly attributable to Citizen Science project). Data from Park rangers provided by the GPNP were included in this category because they were derived from random observations made by Park rangers during their surveillance paths and did not belong to regular sampling activities.
- *Sampling*, structured data collection adhering to rigorous protocols and standardised methodologies that enable quantitative or semi‐quantitative analysis. This includes carefully designed sampling plans for selecting locations and thorough training of operators in both butterfly identification and sampling techniques. This source includes all data recorded by people trained in butterfly identification. This category includes all available data from sampling by researchers belonging to the Zoological Laboratory of the University of Turin and GPNP (Bassano et al. 2021). This includes monitoring for Habitats Directive (92/43/EEC) submitted to the European Commission and data on Parnassius apollo (unpublished data). The GPNP provided all the monitoring data in their possession, which mainly derived from the “Animal Biodiversity Monitoring Project” (unpublished data), but also from spot monitoring carried out for impact assessments (e.g. construction of “Water and Biodiversity Centre ‐Rovenaud”).

### 2.3 Potential Extinction Upon Time Series (PETS)

To establish the potential extinction of the butterfly community in the Aosta Valley, we used the R package Potential Extinction upon Time Series index (PETS; (Labadessa et al. 2021; van Tongeren et al. 2023), freely available at: https://github.com/leondap/pets). The input data needed by the algorithm for each record were (i) the species name, (ii) years of occurrence, and (iii) data sources, while the output returned includes the community's extinction potential percentage, the list of species sorted by last record date, and a graph showing the persistence and absence of each species.

Extinction risk evaluated through PETS is a modified version of the extinction rate formula (Pimm et al. 2014) adapted to continuous data (time since last observation) instead of binary data (extinction versus survival). The proportion of years in which the species is absent on the total sampling period, calculated as (last year sample (2022) – last year occasion)/(last year sample (2022) – first record) that varies between [0, 1) and reflects the fraction of time a species has gone unobserved since the first record. To understand the extinction potential of butterfly communities, we calculated PETS values for the entire dataset, and we calculated the PETs index for (i) all available sources and for (ii) all data except those of the ‘Citizen Science’ source (similarly to van Tongeren et al. 2023).

### 2.4 Spatial Distribution in Relation to Source Categories

To compare the spatial distribution of records of each data source, we projected each georeferenced datum onto a 10 km × 10 km resolution grid (42 cells for the entire region) and visualised the point density within each cell using colour gradations. Data extraction and cartographic operations were carried out with QGIS (v. 3.16.7).

### 2.5 Statistical Analysis

All analyses were carried out in the R (v. 4.2.2) statistical environment (R Core Team 2022).

For Generalised Linear Mixed Models (GLMMs), we used the glmmTMB package (Brooks et al. 2017). Each Generalised Additive Model (GAM) was fitted using the MGCV package (Wood 2011). For the post hoc tests, we used the MULTCOMP package (Hothorn et al. 2008); for graphs, we used the VISREG package (Breheny and Burchett 2017).

#### 2.5.1 Temporal Distribution by Source Category Analysis

To investigate the distribution over time and which of the three source categories had contributed more to the dataset, we modelled observations (calculated as the number of records per species, year and source category) and species (calculated as species richness per year) in two GAMs (GAM1 and GAM2). We plotted variables related to butterfly communities (observations and species richness) versus all parameters, allowing us to establish which variables were linear and which were nonlinear. Thus, years, as a smooth continuous variable, and sources, as a categorical variable, were used as explanatory variables. Considering that distribution over years could be different in relation to source, we added the interaction Years and Source as smooth terms (Observations/Sp_richness ~ s(Years, by = Source)). Considering that the data of observations and species richness are over/under dispersed, we specified a negative binomial family distribution of errors.

#### 2.5.2 Ecological Trait and Source Analysis

To understand which species traits affect representativeness in relation to sources, we modelled the number of observations (sum of all records over years and separated per source categories and species) in GLMM using interaction terms of sources and voltinism (Sources × Voltinism) as categorical explanatory variables, sources and altitudinal range, wingspan, and log‐transformed number of host plant genera as continuous explanatory variables (Source × Altitudinal_range, Source × Wingspan, Source × Host_plant_Genera). The butterfly species (1|Species) were added as a random factor. Considering that the data of observations are over/under‐dispersed, we specified a negative binomial family distribution of errors.

To understand if local extinction risk can be linked to ecological traits, we obtained potential extinction for each species for each source (data derived from PETS). These values were used in GLMMs using the same predictors as the previous model. Butterfly species were added as a random factor. Considering that the dataset presents numbers that range between [0, 1), we specified the Beta family with zero‐inflation.

#### 2.5.3 Ecological Trait Analysis

To understand overall which species traits affect representativeness, we modelled the number of observations (sum of all records over years and separated per species but not per source) in GLMM using voltinism as a categorical explanatory variable, and altitudinal range, wingspan, and log‐transformed number of host plant genera of host plants as continuous explanatory variables. The butterfly genera (1|Genera) were added as a random factor to account for phylogenetic autocorrelation. Considering that the data of observations are over/under dispersed, we specified a negative binomial family distribution of errors.

To understand if local extinction risk can be linked to ecological traits, we used potential extinction calculated for each species in GLMMs using the same predictors as the previous model. The butterfly genera (1|Genera) were added as a random factor to account for phylogenetic autocorrelation. Considering that the dataset presents numbers that range between [0, 1), we specified the Beta family with zero‐inflation.

## 3 Results

In total, the database counts 30,589 records, of which 238 were excluded due to the absence of the year (e.g., collections or faunistic literature), resulting in 30,351 records. There were 173 verified species over 118 years, with the first record in 1825 and the last in 2022. Among the 30,351 records, we were able to georeference 30,320 records with different resolutions according to the available information: 15,547 with the highest resolution (1 km × 1 km), 8102 with a medium resolution (3 km × 3 km), and 6671 with the coarsest resolution (10 km × 10 km) (Table S1). Of the 31 whose resolution is missing, only reporting the datum for the region, 3 belong to Citizen Science, and 28 belong to Literature and Collections.

Overall in the database, all species are present in the Literature and Collections category, while in the Citizen Science category, 13 species are missing (7.5%) and in the Sampling category, 42 species (24.7%; Figure S1A). 10 species have been recorded last time before 1996 and are not recorded either in citizen science or sampling categories, and they encompass the highest contribution to PETS (Table S2).

### 3.1 Data Source Analysis, Temporal and Spatial Distribution

To assess the data distribution among the different sources, we first checked the number of records belonging to each of the three categories, as a proxy for reporting efficiency. We found that 16,433 of 30,351 (54.1%) belong to Citizen Science; 7957 (26.2%) come from Literature and Collections; finally, from Sampling, we obtained 5961 records (19.7%; Supporting Information S1).

**FIGURE 1:**
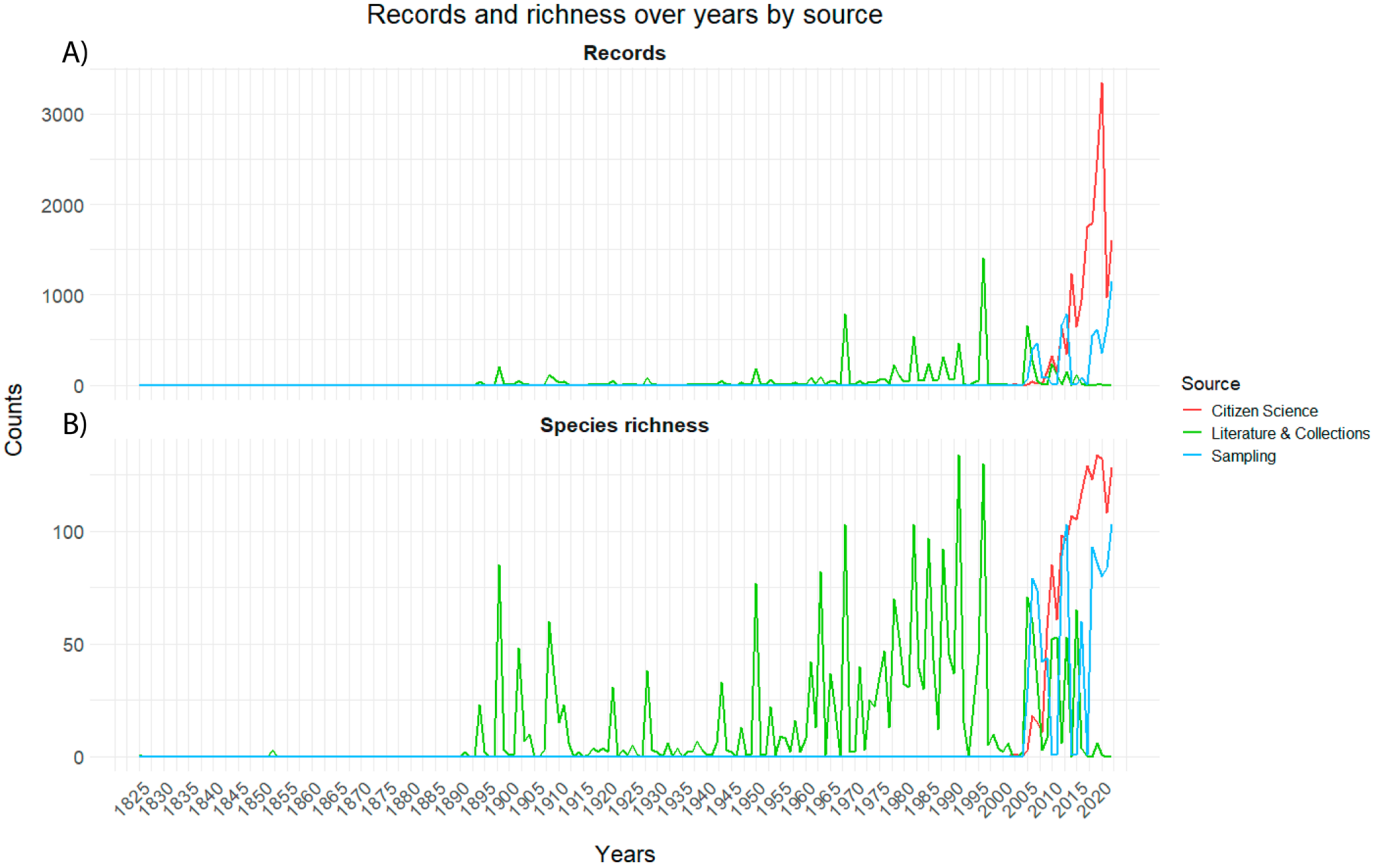
Lineplot with (A) distribution of records and (B) recorded species richness over the years in relation to different source categories. Graphs were drawn using the ‘ggplot2’ package in R.

The trends of records and species richness over the years differ among sources (Figure 1). Specifically, records of Citizen Science were around zero until 2000 due to the absence of data (GAM1; deviance explained = 79.5%; Table S4), then they increased from around 2000 to 2022 with small confidence intervals (edf = 5.839, *χ* ^2^ = 316.8, *p* < 0.001***), while Sampling shows a similar trend (edf = 5.248, *χ* ^2^ = 304.6, *p* < 0.001***). Literature & Collections (edf = 8.850, *χ* ^2^ = 457.2, *p* < 0.001***) showed an oscillating trend until the end of the 1900s, then declined over the last 30 years (Figure 1A). Similarly to results for records, species richness (GAM2; deviance explained = 78.6%; Table S5) recorded by Citizen Science and Sampling is low until the beginning of 2000 (some users uploaded old images with collection date), then it increased from around 2000 to 2022 with small confidence intervals (Citizen Science: edf = 5.066, *χ* ^2^ = 271.9, *p* < 0.001***; Sampling: edf = 4.776, *χ* ^2^ = 252.8, *p* < 0.001***). Literature & Collections (edf = 8.216, *χ* ^2^ = 376.6, *p* < 0.001***) fluctuates until the end of the 20th century and then declines in the last 30 years (Figure 1B).

Before 2005, there were 6328 records (20.8%), and 24,023 (79.2%) after 2005 (Figures S2 and S3). As expected, all pre‐2005 records are from the Literature & Collections category (mainly literature and museum collections). After 2005, the Literature & Collections category included only 6.8% (1631, including 25 “Year assigned” records) of the total 24,023, compared with 24.8% (5961, including 19 “Year assigned” records) from Sampling and 68.4% (16,431) from Citizen Science. The latter, in particular, started to contribute exponentially since 2009. The number of species identified before 2005 turned out to be 171 out of 173 (98.8%), with *Cacyreus marshalli* and *Euphydryas intermedia* as species missed, since their first records are from 2007 and 2008 in Literature & Collections, respectively.

**FIGURE 2:**
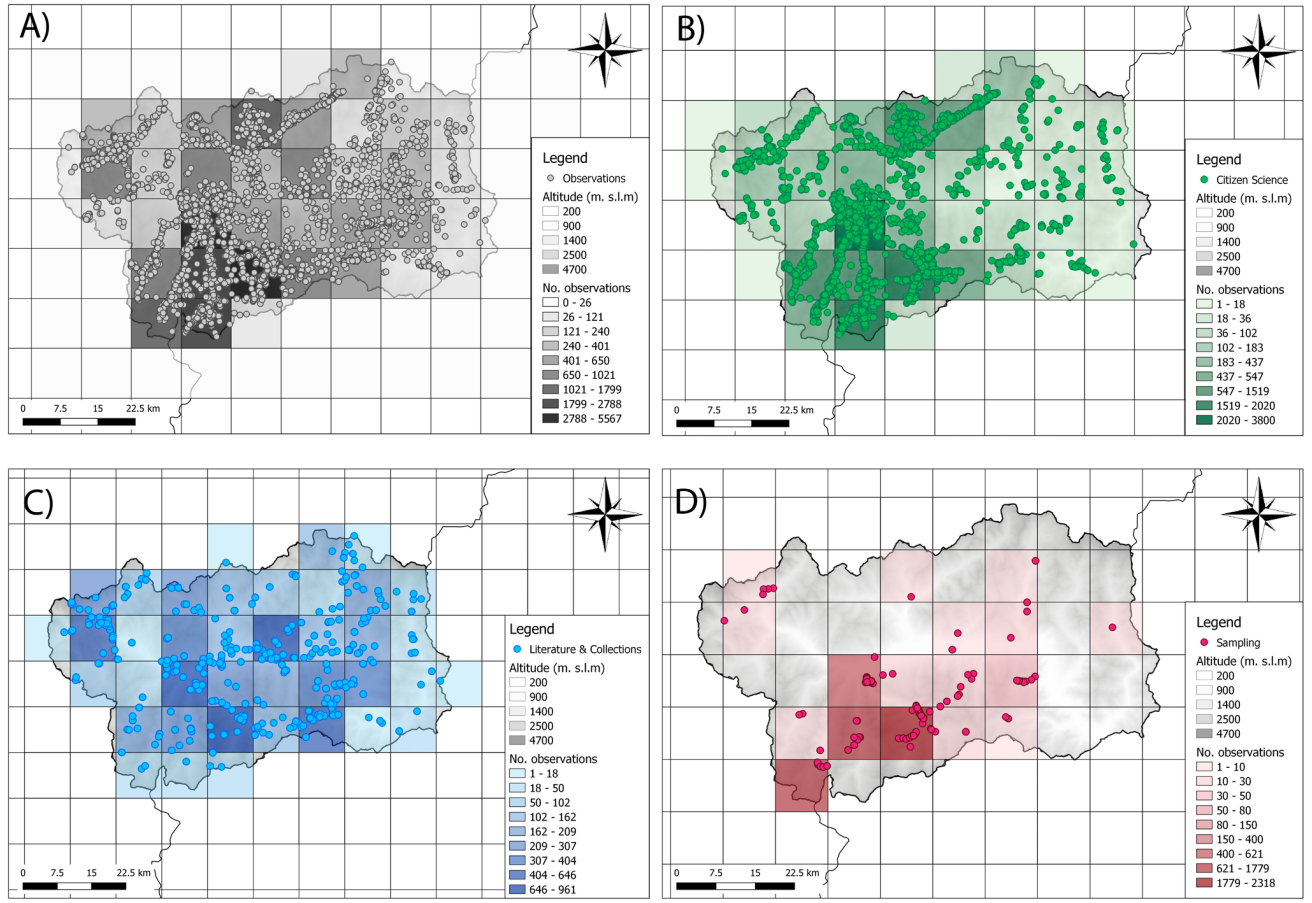
Spatial distribution among the three sources: All observations in grey (A); Citizen Science in green (B); Literature & Collections in blue (C); and Sampling in red (D). Each cell carries a graduated colour according to the density of dots present (light = few dots; dark = many dots). Background grid of 10 km × 10 km.

Projecting the overall data, indistinctly from the sources, there were a large concentration of points in the southwestern part of the region, an area largely included within the Gran Paradiso National Park (Figure 2A). We found that Citizen Science covered a larger area than the other two sources, with 40 cells out of 42 total (95% of region cells; 4000 km^2^) with recorded presence compared to 39 (92% of region cells; 3900 km^2^) for Literature and Collections and 17 (40% of region cells; 1700 km^2^) for Sampling (Figure 2B–D; details on the resolution for each source in Table S1).

### 3.2 Species Potential Extinctions and Representativeness in Relation to Years of Occurrence

**FIGURE 3:**
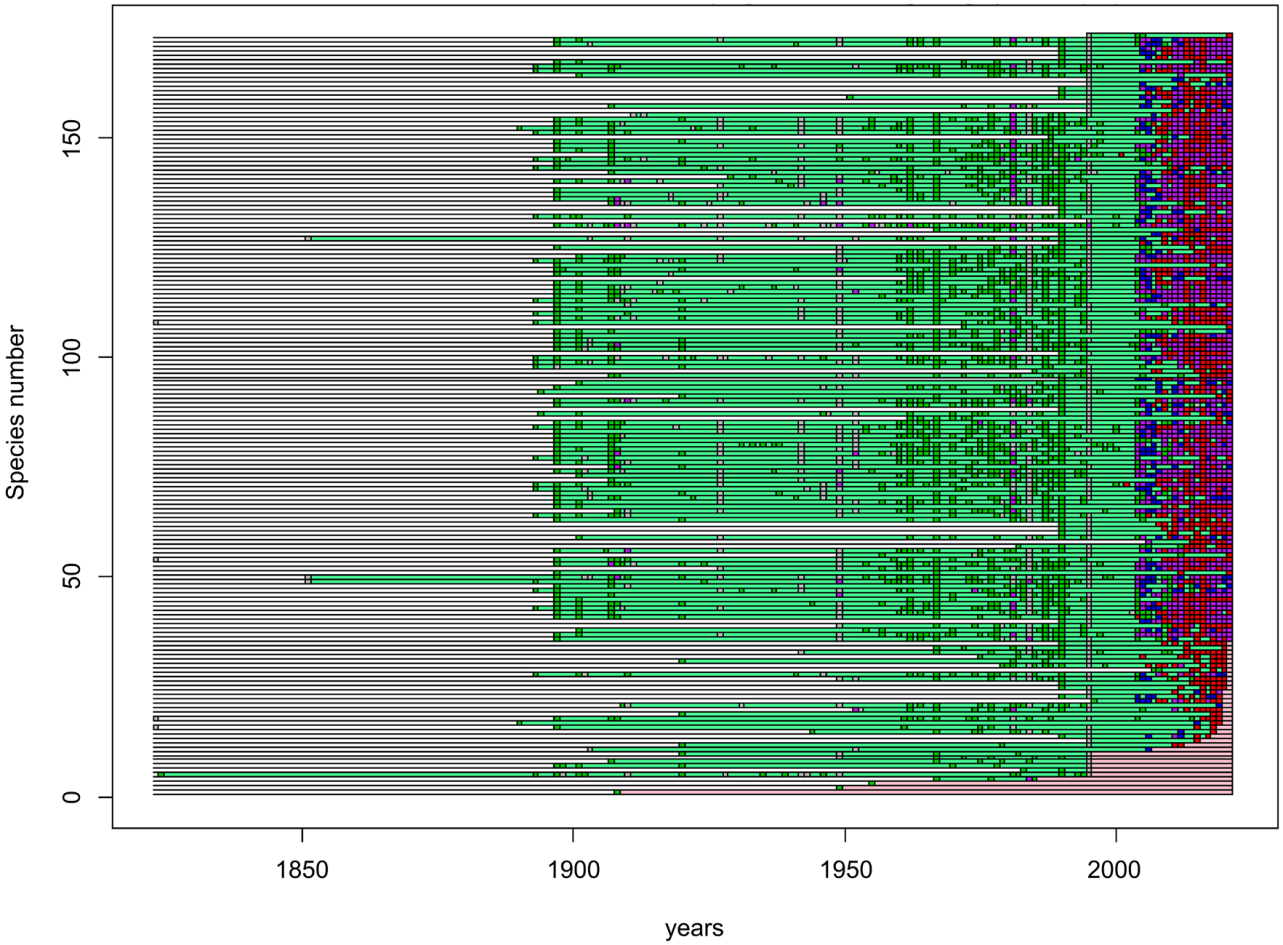
Results from PETs analysis. Each row on the *Y*‐axis represents a species while the *X*‐axis shows the years of records marked by coloured squares, where the colour indicates the type of data source: Red = Citizen Science; green = Literature & Collections; blue = Sampling; grey = Year assigned; purple = plural sources. Green bars indicate persistence, pink bars the absence of the species from the last record to the last year. Species with more recent records are shown at the top, while older last records are shown at the bottom.

The first result returned by PETS is a representation of the persistence or apparent disappearance of species over the years (Figure 3). From 2005, an increase in records is evident, and relatively few species have not been recorded in 2022 (pink lines in Figure 3). In fact, the regional extinction potential, which is calculated as the ratio of the length of time since the last record (red lines) divided by the time since the first record (red + green lines), is 2.9% for Aosta Valley. Excluding the “Citizen Science” category, the regional extinction potential increases to 7.2%.

The species list includes 35 species no longer reported in 2022, of which 23 have been observed in the last 7 years (Table S3). Of the 12 remaining, 2 have been observed in 2007 (*Erebia medusa*) and 2013 (*Colias hyale*) as the last records. Only 10 species have the last occurrences dating back to before 1996 (Table 1). Among those, 3 (*Polygonia egea, Coenonympha glycerion* and *Araschnia levana*) have only one historical record. We have constructed a map of these 10 species, showing their relative elevations and record distribution (Figure S4).

**TABLE 1:** The table shows the voltinism and altitude usually frequented by the species, whose occurrence was reported only before 1996 (species not present in both citizen science and sampling categories).

Species	First obs	Last obs	Univoltine	Elevation (m. a.s.l.)	Habitat
Polygonia egea *	1909	1909	No	Up to 1200	Meadows, large clearings and forest edges
Coenonympha glycerion *	1950	1950	Yes	Up to 2000	Grassy slopes (from dry to damp)
Araschnia levana *	1956	1956	No	Up to 1000	Sparse woodland edges, small clearing
Erebia eriphyle **	1968	1986	Yes	1200–2400	Moist alpine grasslands, shrublands, rocky slopes
*Argynnis pandora*	1825	1996	Yes	Up to 1200	Shrublands and clearings (usually dry)
Leptidea juvernica **	1984	1996	Yes	Up to 2000	Wet meadows, floodplains
*Limenitis camilla*	1898	1996	Yes	Up to 1500	Light woods and clearings
*Lycaeides argyrognomon*	1908	1996	No	Up to 1300	Grasslands and dry environments
*Pyronia tithonus*	1950	1996	Yes	Up to 1400	Uncultivated places with brambles, light woodland edge, scrubland
Thecla betulae **	1921	1996	Yes	Up to 1800	Broadleaf woods edges

*Note:* The habitat commonly used in Italy is also highlighted; species with only one report are marked with an asterisk (*), with ten or less than 10 reports are marked with two asterisks (**).

#### 3.2.1 Ecological Trait and Source Analysis

Description of trait variables (i.e., mean, median, min, max, quartiles and counts of NAs) can be found in Table S3. The number of records did not change in relation to sources and ecological traits (see Table S6) but changed with sources (*χ* ^2^ = 113.410, *p* < 0.001***; Figure S5A) and altitudinal range (*χ* ^2^ = 5.849, *p* = 0.016*; Figure S5B; Table S6).

Records were significantly higher for citizen science than for sampling (est. = −1.268, *Z* = −10.174, *p* < 0.001***) and literature (est. = −0.355, *Z* = −2.796, *p* = 0.014*) and were higher for literature than the sampling category (est. = −0.912, *Z* = −6.872, *p* < 0.001***; Figure S5A and Table S7).

**FIGURE 4:**
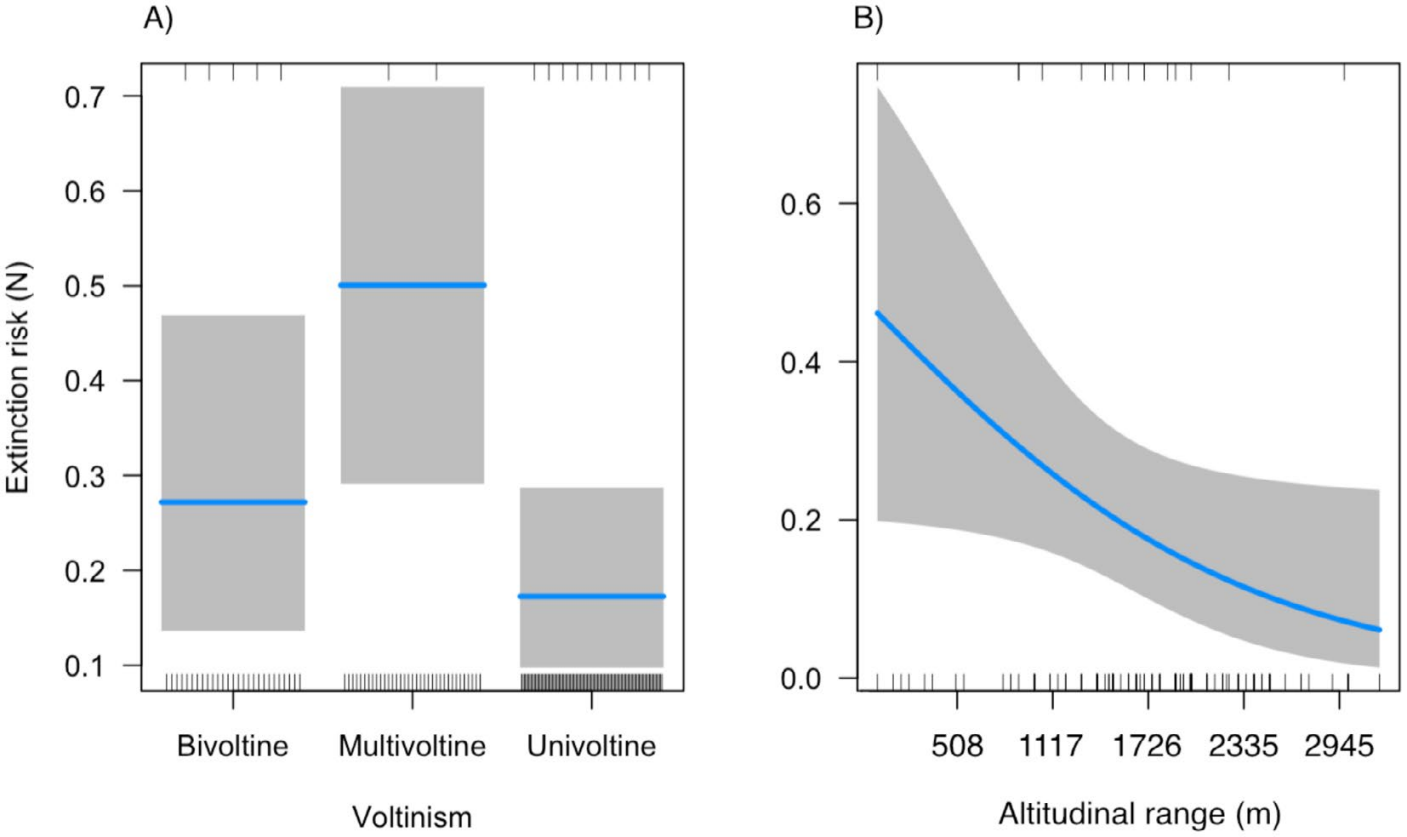
Species extinction probability was significantly higher for multivoltine species than for univoltine ones (A) and decreased with increasing altitudinal range (B). Lines represent the best‐fit models; shadows are the 95% confidence interval. Graphs were drawn using the ‘visreg’ package in R.

Species extinction probability did not change in relation to the interaction between sources and ecological traits (Table S8) but changed significantly among sources (*χ* ^2^ = 9.918, *p* = 0.007**; Figure S5C and Table 8). Indeed, it depends directly on the year of the species' first and last observations; consequently, in the source category comprising fewer recent records, the species extinction probability appears higher. Literature showed the highest value (est. = 0.731, *Z* = 3.239, *p* = 0.003**) followed by sampling (est. = 0.571, *Z* = 2.513, *p* = 0.032*) and citizen science (Figure S5C and Table 9). Moreover, species extinction probability decreased with increasing altitudinal range (*χ* ^2^ = 8.124, *p* = 0.004**; Figure 4E; Figure S5D and Table S8).

### 3.3 Ecological Trait Analysis

Species records did not change in relation to ecological traits (Table 10) but extinction probability changed in relation to voltinism (*χ* ^2^ = 7.701, *p* = 0.021*; Figure 4A) and altitudinal range (*χ* ^2^ = 3.924, *p* = 0.048*; Figure 4B; Table S11). Multivoltine species have a higher extinction probability than univoltine ones (est. = −1.569, *Z* = −2.748, *p* = 0.016*; Figure 4A; Table S12).

## 4 Discussion

### 4.1 Data Source Analysis and Spatio‐Temporal Distribution

The data source analysis showed that more than 54% of the records in our database were composed of citizen science data, confirming the importance of citizen or volunteer involvement in research (Labadessa et al. 2021; Sanderson et al. 2021; Fontaine et al. 2022). However, the large number of records does not correspond to increased species richness. Indeed, Literature contributed to 26% of all records, included all species of the region, while 13 and 42 species have not been recorded in citizen science and sampling categories, respectively. Some species not recorded recently either in citizen science or sampling categories have likely suffered a recent strong decline and local extinctions; for example, *Argynnis pandora* and Araschnia *levana* (Bonelli et al. 2011).

Spatio‐temporal trends of records and species richness over time differed significantly between sources. Citizen science from around 2008 started to increase while sampling and literature had trends with several peaks and flows, likely due to different efforts over the years due to different funding and/or scientific interests (see for example, Viterbi et al. 2013). Moreover, we recorded a general decline of species and records in literature in recent years, and this might be linked to an increased digital approach that uses online tools to record species (e.g., Fontaine et al. 2021) and to the current scientific trends, focusing more on single species (such as threatened or protected or charismatic—*Maculinea* spp. – species). However, the spatial distribution of data differed between sources. Citizen science data are spatially more concentrated in the southern part of the region where the Gran Paradiso National Park is located, while literature is more widespread across the region. Indeed, citizen science data are often biased and more frequent in accessible or more popular areas (e.g., Tiago et al. 2017). Sampling is contributing less and in restricted areas, likely because most of the data was derived from monitoring activities carried out inside the National Park. These results support the evidence that protected areas can greatly contribute to biodiversity knowledge and scientific research by attracting nature‐based tourism, which provides citizen science data, and by promoting monitoring projects.

### 4.2 Species Potential Extinctions

The potential extinction of the butterfly community in Aosta Valley was quite low, summing to 2.9%. However, if we remove the contributions of Citizen Science, the extinction risk increases to 7.2% since 54% of the total records come from this source. Moreover, citizen science records are usually very recent and thus have great power in confirming the current presence of many species (van Tongeren et al. 2023). This underscores the substantial impact that Citizen Science has on the data, illustrating that its absence could lead to a considerable overestimation of the potential extinction risk.

The analysis of species potential extinctions helped us to value two important aspects: the first one is a positive result, such as the fauna of the Aosta Valley showing most species reconfirmed over time (conversely to what is happening in many other parts of Europe, e.g. Warren et al. 2021). The second one is related to the species no longer seen (Figure S2). These should not automatically be considered extinct. They represent instead a wake‐up call for the need to actively search for them in order to determine whether they have indeed disappeared or not.

Since 1996, 10 species have not been recorded in the region (Table 1). Among these, 3 species have been recorded only once and in one single locality in the region: *Coenonympha glycerion* (recorded in 1950), *Polygonia egea* (recorded in 1909) and *Araschnia levana* (recorded in 1956). Consequently, their known presence in the Aosta Valley can be considered highly localised in space and/or sporadic in time and based only on a few historical records. *Coenonympha glycerion* in all the Italian Alps is characterised by few, small, isolated populations and has already been recorded to have reduced significantly (Bonelli et al. 2022). *Araschnia levana* is extremely rare in Italy and assessed as Endangered (EN) at the national level (Bonelli et al. 2018) having reduced its distribution in the country by 90% during the last 50 years (Bonelli et al. 2011). *Polygonia egea* is also rare and localised in northern Italy, and it inhabits mainly dry grasslands or clearings, which are uncommon in the Aosta Valley. It is therefore very likely that these 3 species are no longer present in the region or that single, vagrant individuals not forming stable populations have been recorded in the past.

Other 7 species are not recorded anymore in Aosta Valley, and we cannot distinguish if they are locally extinct or difficult to observe. *Erebia eriphyle* is more widespread but always localized in the Aosta Valley (as in the rest of the Italian Alps) and morphologically similar to some common congenerics, and consequently, it is less likely to be recorded, especially by citizens. *Lycaeides argyrognomon* and *Pyronia tithonus* can be preferably found at low elevations (up to 1200 m), and for this reason, might have accumulated few records in the Aosta Valley, predominantly mountainous. *Limenitis camilla* and *Thecla betulae* are both ecotonal species that might have been negatively affected by plant encroachment and habitat reduction (Bonelli et al. 2018). Moreover, both are forest species, mainly flying near tree canopies, making them difficult to record. *Argynnis pandora* could also have suffered from plant encroachment: it is known to have a localized distribution in the Aosta Valley, in particular, due to its habitat preferences, and has probably reduced its distribution area (Bonelli et al. 2011). It is usually present with low‐density populations in Northern Italy but has been historically recorded by entomologists, especially due to its rarity in the region. It is also a strong flyer, usually fast flying near the tree canopy, and could be easily confounded with the more common *Argynnis paphia* by untrained observers, underlining the need for expert assessment to ascertain its current situation in the Region. Concerning *Leptidea juvernica*, records of this species are not recent essentially because they can be derived exclusively from literature data (Dincă et al. 2013). In fact, without sampling the specimens and analyzing the genitalia and/or genetic sequences, it is impossible to diagnose from *L. sinapis* and *L. reali* (Dincă et al. 2011).

### 4.3 Representativeness in Relation to Functional Traits

We found that different categories (citizen science, literature and sampling) did not differ in record numbers in relation to some specific traits (not significant interaction terms Source × functional traits). Thus, different categories did not record more generalist or larger species as expected (Kamp et al. 2016; Callaghan et al. 2021). Likely as a consequence of this, neither extinction risk changed in relation to sources and functional traits (interaction term).

The number of records in our dataset and extinction risk are affected by the source (with the highest contribution by citizen science) and altitudinal range. Some species, such as *Polyommatus humedasae* (Piccini, Pellegrino, et al. 2024; Piccini, Pollo, et al. 2024) or *Parnassius apollo*, have several records because of their popularity (Goldstein et al. 2024); indeed larger (van Tongeren et al. 2023) and charismatic species attract much funding (Mammola et al. 2020) and attention by a broad public (Sumner et al. 2018).

Overall extinction possibility, evaluated through the PETS algorithm, has been related to voltinism and the altitudinal range. These results are likely not driven by a shift away from literature/collections to citizen science data and any potential shift in types of species that are reported. Univoltine species have higher records and lower extinction probabilities. This might be related to the fact that some charismatic univoltine species have attracted more conservation efforts (see for example protected species). However, it has been shown that climate change can create developmental traps in multivoltine species with the emergence of “lost generations” as hypothesized to explain the decline of Lasiommata megera in Northern Europe (Van Dyck et al. 2015). Moreover, species with smaller altitudinal ranges are less likely to be observed in recent years and thus have a higher extinction probability. Undoubtedly, climate change has virtually influenced the butterfly community, shrinking the altitudinal distribution of many Alpine specialist taxa to smaller and less reachable mountain areas. Moreover, the response between plants and butterflies has been different, thus increasing the mismatch between them, mostly when specialist taxa are involved (Cerrato et al. 2016; Piccini, Pittarello, Di Pietro, et al. 2022).

## 5 Conclusions

Our study shows that in the Aosta Valley, the frequency of species observations depends on their popularity and their polyphagia, which also determines their distribution. Conversely, the recorded extinction potential is mainly linked to the altitudinal range of distribution. Indeed, several species no longer reported since 1996 are often of low to medium altitude with limited altitudinal range, and in some cases, are difficult to identify.

This study presents the first comprehensive, regional database of Aosta Valley butterflies that includes citizen, park staff, literature, and researcher sampling data. It is easily accessible, and it might be useful for future investigations of citizens, researchers, and policymakers. In this way, data collected by citizens and researchers have been analyzed and results interpreted by researchers, and these results are returned to citizens in a continuous proficient dialogue between scientists and citizens. Indeed, for those species not recently recorded (such as *Pyronia tithonus*), easy to identify and of low to medium altitudes, volunteers of citizen science can help provide new data on these species. This creates a virtuous cycle in which citizens become essential in searching for these missing species, effectively bridging the gap between citizen science and academic research. Other species more complex to find and identify can be searched for by experts. Their absence needs to be confirmed by specific surveys (e.g., *Polygonia egea, Coenonympha glycerion, Araschnia levana, Leptidea juvernica, Erebia eriphyle* and *Plebejus argyrognomon* ) and might be essential for ecological and conservation purposes. Thus, our results highlight the complementarity of monitoring and Citizen Science data, in particular in terms of spatial coverage (Hadj‐Hammou et al. 2017; Mandeville et al. 2022) and the importance of data integration to achieve effective biodiversity monitoring (Kühl et al. 2020).

Unstructured citizen science is indeed very useful for medium‐to large‐scale distribution analyses. More in‐depth monitoring efforts carried out by professionals, with higher spatial resolution, precise objectives (e.g., evaluation of management actions, spatial and population dynamic of target species), and long‐term repetitions are usually more localized in space but essential to provide more detailed information (e.g., Kamp et al. 2016).

## Author Contributions

**S. Alberti:** conceptualization (supporting), data curation (equal), formal analysis (equal), investigation (equal), methodology (supporting), resources (equal), visualization (equal), writing – original draft (equal), writing – review and editing (equal). **A. Pollo:** project administration (lead), writing – review and editing (equal). **C. Cerrato:** data curation (supporting), resources (supporting), writing – review and editing (equal). **R. Viterbi:** resources (supporting), writing – review and editing (equal). **E. Balletto:** resources (equal), writing – review and editing (equal). **L. Dapporto:** formal analysis (supporting), investigation (supporting), methodology (equal), supervision (equal), writing – review and editing (equal). **S. Bonelli:** funding acquisition (lead), writing – review and editing (equal). **I. Piccini:** conceptualization (equal), data curation (equal), formal analysis (equal), investigation (equal), methodology (equal), supervision (lead), visualization (supporting), writing – original draft (equal), writing – review and editing (equal).

## Conflicts of Interest

The authors declare no conflicts of interest.

## Supporting information


**Figure S1.** Regional observations efficiency in the three groups (A); and richness efficiency in the three groups (B).
**Figure S2:** Histograms showing number of records per year. highlighting the temporal distribution of data.
**Figure S3:** Highlighting the temporal distribution of regional data. breakdown of pre‐ and post‐2005 data for the three groups + the “Year assigned” category.
**Figure S4:** Spatial distribution and relative altitude of regional species no longer present since 1996. GPNP is Gran Paradiso National Park. while MANP is Mont Avic Natural Park The map was drawn using QGIS.
**Figure S5:** Observations changed in relation to source categories (A) and increased with increasing altitudinal range (B). Extinction risk changed in relation to source category (C) and decreased increasing the altitudinal range (D). Lines represent the best‐fit models, shadows are the 95% confidence interval. Graphs were drawn using the ‘visreg’ package in R.
**Table S1:** List of observations according to spatial resolution and source. Three different spatial resolutions were identified and for each, the number of records is indicated 1 × 1 km: records with resolution higher than 1 km; 3 × 3 km: records with resolution between 1 and 3 km; 10 × 10 km records with resolution between 3 and 10 km. Data with unknown resolution or lower than 10 km have been discarded from this analysis.
**Table S2:** Species not observed per sources. Species no longer reported since 1996 are identified in grey; with only one report with an asterisk (*).
**Table S3:** Summary of trait variable features. Wingspan was calculated as the average of male and female values (from Middleton‐Welling et al. 2020), voltinism and host plant genera were derived from the European database (Middleton‐Welling et al. 2020). The altitudinal range was calculated as the interval between min and max altitude where the species can be found in the Alps (Tolman 2008).
**Table S4:** Results from the GAM1 of records over the years in relation to the sources. Signif. codes: 0 ‘***’ 0.001 ‘**’ 0.01 ‘*’ 0.05 ‘.’ 0.1 ‘’ 1.
**Table S5:** Results from the GAM2 of species richness over the years in relation to the sources. Signif. codes: 0 ‘***’ 0.001 ‘**’ 0.01 ‘*’ 0.05 ‘.’ 0.1 ‘’ 1.
**Table S6:** Results from the GLMM of species records of species per source in relation to functional traits. Signif. codes: 0 ‘***’ 0.001 ‘**’ 0.01 ‘*’ 0.05 ‘.’ 0.1 ‘’ 1.
**Table S7:** Post hoc results from the GLMM without non‐significant interaction terms of species records in relation to sources. Signif. codes: 0 ‘***’ 0.001 ‘**’ 0.01 ‘*’ 0.05 ‘.’ 0.1 ‘’ 1.
**Table S8:** Results from the GLMM of PETS extinction risk divided for source in relation to functional traits. Signif. codes: 0 ‘***’ 0.001 ‘**’ 0.01 ‘*’ 0.05 ‘.’ 0.1 ‘’ 1.
**Table S9:** Post hoc results from the GLMM without non‐significant interaction terms of species records in relation to sources. Signif. codes: 0 ‘***’ 0.001 ‘**’ 0.01 ‘*’ 0.05 ‘.’ 0.1 ‘’ 1.
**Table S10:** Results from the GLMM of species records in relation to functional traits. Signif. codes: 0 ‘***’ 0.001 ‘**’ 0.01 ‘*’ 0.05 ‘.’ 0.1 ‘’ 1.
**Table S11:** Results from the GLMM of species extinction risk in relation to functional traits. Signif. codes: 0 ‘***’ 0.001 ‘**’ 0.01 ‘*’ 0.05 ‘.’ 0.1 ‘’ 1.
**Table S12:** Post hoc results from the GLMM of species extinction risk in relation to ecological traits. Signif. codes: 0 ‘***’ 0.001 ‘**’ 0.01 ‘*’ 0.05 ‘.’ 0.1 ‘’ 1.

## Data Availability

We provide data in an accessible data archive: https://doi.org/10.6084/m9.figshare.28241531.
