# Supplementary material for: Unrecorded Butterfly Species and Potential Local Extinctions: The Role of Citizen Science and Sampling
Source: Ecol Evol. 2025 Feb 17;15(2):e71023. doi: 10.1002/ece3.71023 (PMC11832908; doi:10.1002/ece3.71023)
Supplement: Supplementary file 1 — Figure S1. Regional observations efficiency in the three groups (A); and richness efficiency in the three groups (B). Figure S2: Histograms showing number of records per year. highlighting the temporal distribution of data. Figure S3: Highlighting the temporal distribution of regional data. breakdown of pre‐ and post‐2005 data for the three groups + the “Year assigned” category. Figure S4: Spatial distribution and relative altitude of regional species no longer present since 1996. GPNP is Gran Paradiso National Park. while MANP is Mont Avic Natural Park The map was drawn using QGIS. Figure S5: Observations changed in relation to source categories (A) and increased with increasing altitudinal range (B). Extinction risk changed in relation to source category (C) and decreased increasing the altitudinal range (D). Lines represent the best‐fit models, shadows are the 95% confidence interval. Graphs were drawn using the ‘visreg’ package in R. Table S1: List of observations according to spatial resolution and source. Three different spatial resolutions were identified and for each, the number of records is indicated 1 × 1 km: records with resolution higher than 1 km; 3 × 3 km: records with resolution between 1 and 3 km; 10 × 10 km records with resolution between 3 and 10 km. Data with unknown resolution or lower than 10 km have been discarded from this analysis. Table S2: Species not observed per sources. Species no longer reported since 1996 are identified in grey; with only one report with an asterisk (*). Table S3: Summary of trait variable features. Wingspan was calculated as the average of male and female values (from Middleton‐Welling et al. 2020), voltinism and host plant genera were derived from the European database (Middleton‐Welling et al. 2020). The altitudinal range was calculated as the interval between min and max altitude where the species can be found in the Alps (Tolman 2008). Table S4: Results from the GAM1 of records over the years in relation to the s [file ECE3-15-e71023-s001.docx]

**Supporting Information for:**

**Unrecorded butterfly species and potential local extinctions: the role of citizen science and sampling**

**Running headline: Butterfly extinction risk in relation to citizen science and sampling**

**Supplementary results**

**Table of Contents:**

| **Fig S1** | Regional observations efficiency in the three groups (A); and richness efficiency in the three groups (B). |
| --- | --- |
| **Fig. S2** | Histograms showing tnumber of records per year. highlighting the temporal distribution of data. |
| **Fig. S3** | Highlighting the temporal distribution of regional data. breakdown of pre- and post-2005 data for the three groups + the “Year assigned” category. |
| **Fig. S4** | Spatial distribution and relative altitude of regional species no longer present since 1996. GPNP is Gran Paradiso National Park. while MANP is Mont Avic Natural Park The map was drawn using QGIS. |
| **Fig. S5** | Observations changed in relation to source categories (A) and increased with increasing altitudinal range (B). Extinction risk changed in relation to source category (C) and decreased increasing the altitudinal range (D). Lines represent the best-fit models, shadows are the 95% confidence interval. Graphs were drawn using the ‘visreg’ package in R. |
| **Table S1** | List of observations according to spatial resolution and source. Three different spatial resolutions were identified and for each, the number of records is indicated (1x1 km: records with resolution higher than 1 km; 3x3 km: records with resolution between 1 and 3 km; 10x10 km records with resolution between 3 and 10 km. Data with unknown resolution or lower than 10 km have been discarded from this analysis. |
| **Table S2** | Species not observed per sources. Species no longer reported since 1996 are identified in grey; with only one report with an asterisk (*) . |
| **Table S3** | Summary of trait variable features. Wingspan was calculated as the average of male and female values (from Middleton-Welling et al. 2020), voltinism and host plant genera were derived from the European database (Middleton-Welling et al. 2020). The altitudinal range was calculated as the interval between min and max altitude where the species can be found in the Alps (Tolman 2008) |
| **Table S4** | Results from the GAM1 of records over the years in relation to the sources. Signif. codes: 0 ‘***’ 0.001 ‘**’ 0.01 ‘*’ 0.05 ‘.’ 0.1 ‘ ’ 1. |
| **Table S5** | Results from the GAM2 of species richness over the years in relation to the sources. Signif. codes: 0 ‘***’ 0.001 ‘**’ 0.01 ‘*’ 0.05 ‘.’ 0.1 ‘ ’ 1. |
| **Table S6** | Results from the GLMM of species records of species per source in relation to functional traits. Signif. codes: 0 ‘***’ 0.001 ‘**’ 0.01 ‘*’ 0.05 ‘.’ 0.1 ‘ ’ 1 |
| **Table S7** | Post hoc results from the GLMM without non-significant interaction terms of species records in relation to sources. Signif. codes: 0 ‘***’ 0.001 ‘**’ 0.01 ‘*’ 0.05 ‘.’ 0.1 ‘ ’ 1 |
| **Table S8** | Results from the GLMM of PETS extinction risk divided for source in relation to functional traits. Signif. codes: 0 ‘***’ 0.001 ‘**’ 0.01 ‘*’ 0.05 ‘.’ 0.1 ‘ ’ 1 |
| **Table S9** | Post hoc results from the GLMM without non-significant interaction terms of species records in relation to sources. Signif. codes: 0 ‘***’ 0.001 ‘**’ 0.01 ‘*’ 0.05 ‘.’ 0.1 ‘ ’ 1 |
| **Table S10** | Results from the GLMM of species records in relation to functional traits. Signif. codes: 0 ‘***’ 0.001 ‘**’ 0.01 ‘*’ 0.05 ‘.’ 0.1 ‘ ’ 1 |
| **Table S11** | Results from the GLMM of species extinction risk in relation to functional traits. Signif. codes: 0 ‘***’ 0.001 ‘**’ 0.01 ‘*’ 0.05 ‘.’ 0.1 ‘ ’ 1 |
| **Table S12** | Post hoc results from the GLMM of species extinction risk in relation to ecological traits. Signif. codes: 0 ‘***’ 0.001 ‘**’ 0.01 ‘*’ 0.05 ‘.’ 0.1 ‘ ’ 1 |

**Below are additional results carried out for the region Aosta Valley**

For the analyses carried out on the regional database of Aosta Valley we present some additional information.
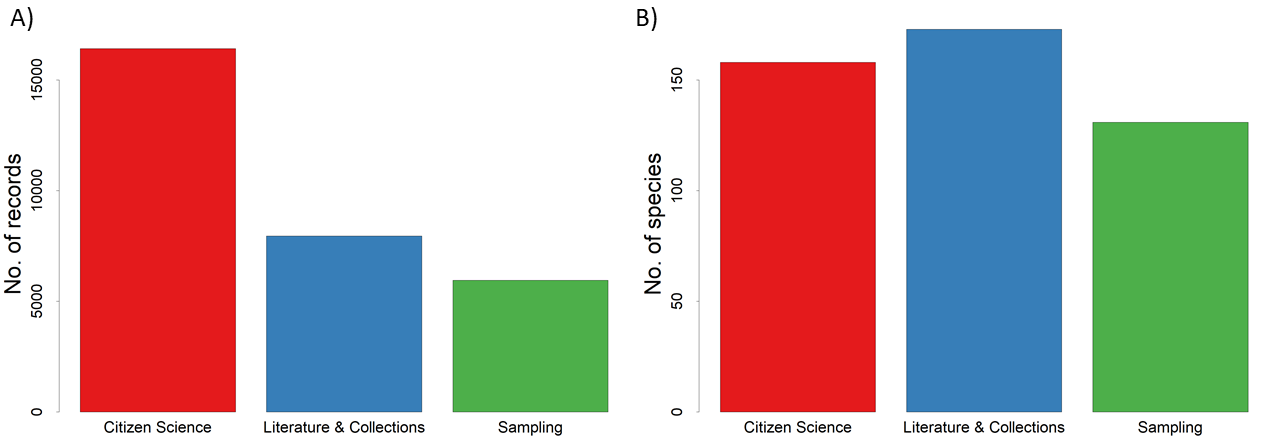


**Fig. S1.** Regional observations efficiency in the three groups (A); and richness efficiency in the three groups (B).


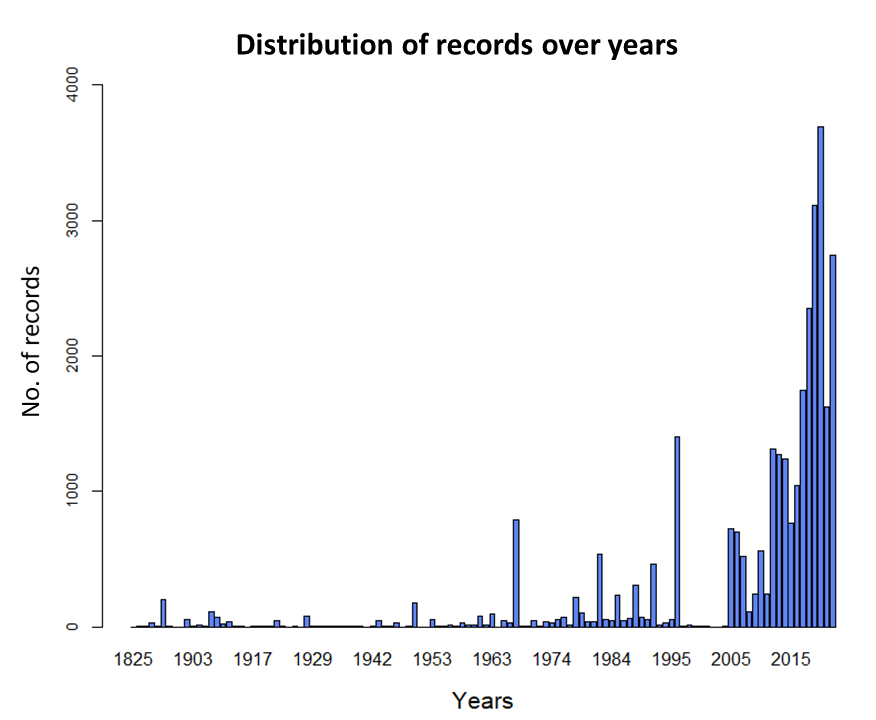


**Fig. S2.** Histogram showing the number of records per year, highlighting the temporal distribution of data.

**
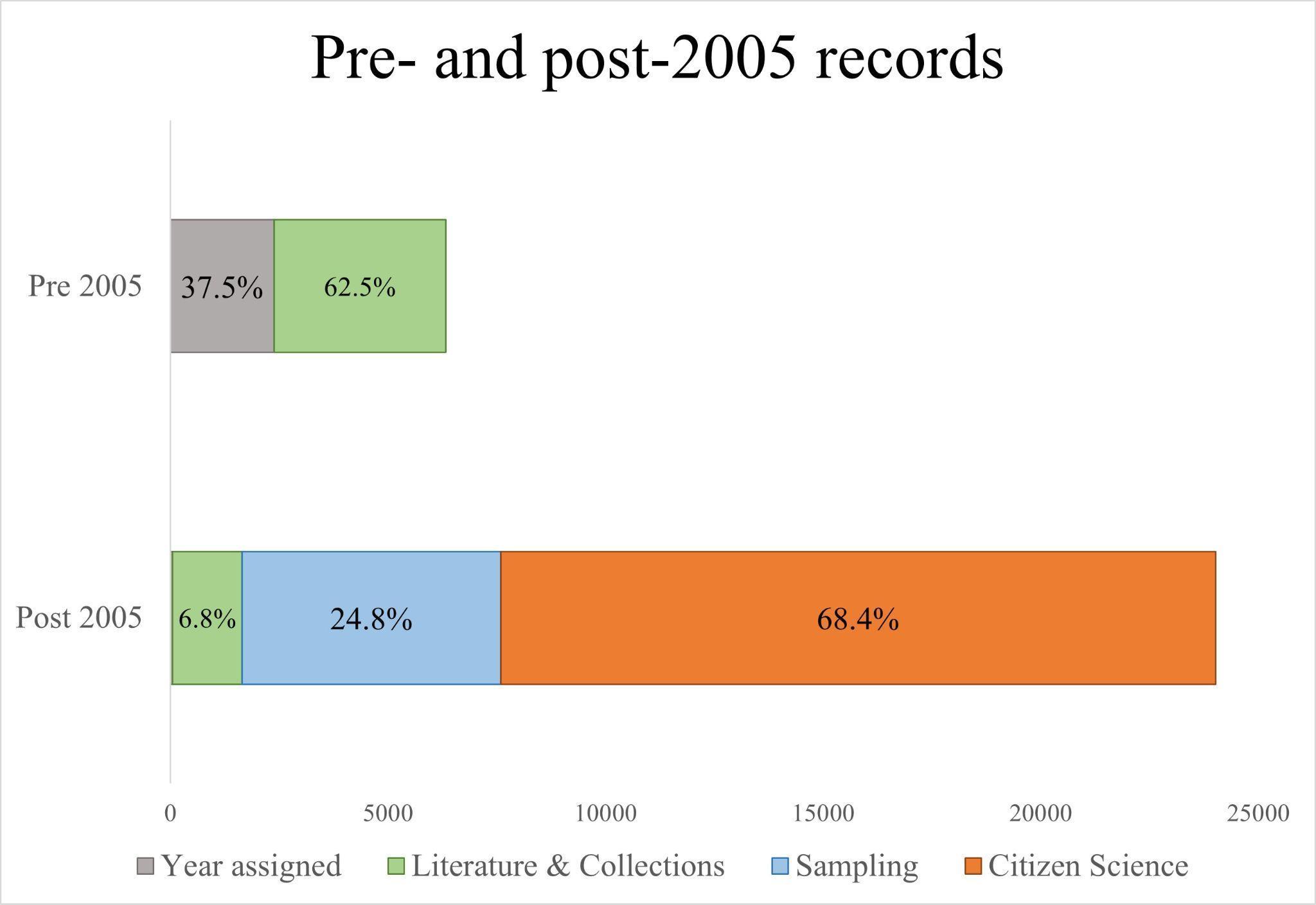
**

**Fig. S3.** Highlighting the temporal distribution of regional data. Breakdown of pre- and post-2005 data for the three groups + the “Year assigned” category.


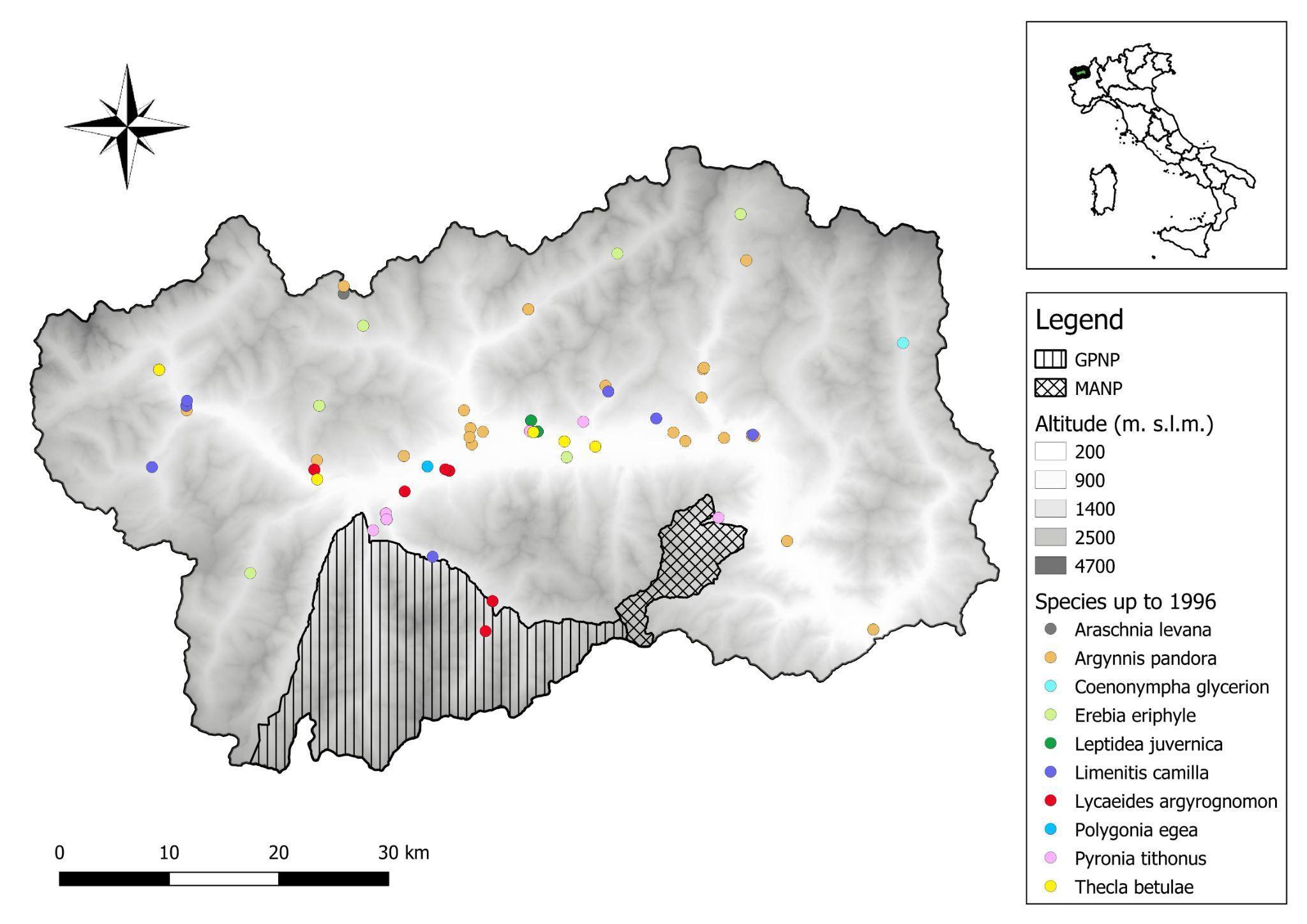


**Fig. S4.** Shows the spatial distribution and relative altitude of regional species that have been absent since 1996. GPNP stands for Gran Paradiso National Park while MANP stands for Mont Avic Natural Park. The map was drawn using QGIS.

**Table S1.** List of observations according to spatial resolution and source. Three different spatial resolutions were identified and for each, the number of records is indicated (1x1 km: records with resolution higher than 1 km; 3x3 km: records with resolution between 1 and 3 km; 10x10 km records with resolution between 3 and 10 km. Data with unknown resolution or lower than 10 km have been discarded from this analysis.

| Resolution | Source | No. observations |
| --- | --- | --- |
| 1 km x 1 km | Citizen Science | 14.385 |
| 1 km x 1 km | Literature & Collections | 232 |
| 1 km x 1 km | Sampling | 930 |
| 3 km x 3 km | Citizen Science | 237 |
| 3 km x 3 km | Literature & Collections | 2.835 |
| 3 km x 3 km | Sampling | 5.030 |
| 10 km x 10 km | Citizen Science | 1.808 |
| 10 km x 10 km | Literature & Collections | 4.862 |
| 10 km x 10 km | Sampling | 1 |

**Table S2.** Species not observed per sources. Species no longer reported since 1996 are identified in grey; with only one report with an asterisk (*) .

| **SPECIES** | **Citizen Science** | **Sampling** | **First obs** | **Last obs** | **Extinction risk** |
| --- | --- | --- | --- | --- | --- |
| *Araschnia levana** | No_observations | No_observations | 1956 | 1956 | 0.99 |
| *Argynnis pandora* | No_observations | No_observations | 1825 | 1996 | 0.13 |
| *Coenonympha glycerion** | No_observations | No_observations | 1950 | 1950 | 0.99 |
| *Erebia eriphyle* | No_observations | No_observations | 1968 | 1986 | 0.65 |
| *Leptidea juvernica* | No_observations | No_observations | 1984 | 1996 | 0.67 |
| *Limenitis camilla* | No_observations | No_observations | 1898 | 1996 | 0.21 |
| *Lycaeides argyrognomon* | No_observations | No_observations | 1908 | 1996 | 0.23 |
| *Polygonia egea** | No_observations | No_observations | 1909 | 1909 | 0.99 |
| *Pyronia tithonus* | No_observations | No_observations | 1950 | 1996 | 0.36 |
| *Thecla betulae* | No_observations | No_observations | 1921 | 1996 | 0.25 |
| *Erebia medusa* | No_observations |  | 1904 | 2007 | 0.13 |
| *Leptidea sinapis* | No_observations |  | 1898 | 2022 | 0.00 |
| *Melitaea aurelia* | No_observations |  | 1910 | 2020 | 0.02 |
| *Apatura iris* |  | No_observations | 1991 | 2021 | 0.03 |
| *Aphantopus hyperantus* |  | No_observations | 1950 | 2022 | 0.00 |
| *Aricia agestis* |  | No_observations | 1898 | 2022 | 0.00 |
| *Boloria dia* |  | No_observations | 1898 | 2022 | 0.00 |
| *Brenthis hecate* |  | No_observations | 1950 | 2022 | 0.00 |
| *Carcharodus alceae* |  | No_observations | 1991 | 2022 | 0.00 |
| *Carterocephalus palaemon* |  | No_observations | 1991 | 2021 | 0.03 |
| *Colias hyale* |  | No_observations | 1921 | 2013 | 0.09 |
| *Cupido osiris* |  | No_observations | 1908 | 2022 | 0.00 |
| *Erebia gorge* |  | No_observations | 1898 | 2020 | 0.02 |
| *Erebia meolans* |  | No_observations | 1898 | 2019 | 0.02 |
| *Erebia pluto* |  | No_observations | 1963 | 2022 | 0.00 |
| *Erebia pronoe* |  | No_observations | 1921 | 2020 | 0.02 |
| *Erebia tyndarus* |  | No_observations | 1953 | 2020 | 0.03 |
| *Euphydryas intermedia* |  | No_observations | 2008 | 2021 | 0.07 |
| *Favonius quercus* |  | No_observations | 1921 | 2022 | 0.00 |
| *Hipparchia hermione/fagi* |  | No_observations | 1986 | 2022 | 0.00 |
| *Iolana iolas* |  | No_observations | 1980 | 2021 | 0.02 |
| *Kanetisa circe* |  | No_observations | 1983 | 2022 | 0.00 |
| *Lampides boeticus* |  | No_observations | 1921 | 2021 | 0.01 |
| *Libythea celtis* |  | No_observations | 1973 | 2022 | 0.00 |
| *Limenitis populi* |  | No_observations | 1976 | 2021 | 0.02 |
| *Lycaena tityrus* |  | No_observations | 1898 | 2022 | 0.00 |
| *Maculinea rebeli* |  | No_observations | 1943 | 2021 | 0.01 |
| *Melitaea parthenoides* |  | No_observations | 1898 | 2022 | 0.00 |
| *Nymphalis polychloros* |  | No_observations | 1898 | 2022 | 0.00 |
| *Pyrgus armoricanus* |  | No_observations | 1996 | 2016 | 0.22 |
| *Satyrium ilicis* |  | No_observations | 1902 | 2022 | 0.00 |
| *Satyrium w-album* |  | No_observations | 1968 | 2021 | 0.02 |
| *Scolitantides orion* |  | No_observations | 1898 | 2022 | 0.00 |
| *Thymelicus acteon* |  | No_observations | 1991 | 2021 | 0.03 |
| *Zerynthia polyxena* |  | No_observations | 1996 | 2022 | 0.00 |

**Table S3.** Summary of trait variable features. Wingspan was calculated as the average of male and female values (from Middleton-Welling et al. 2020), voltinism and host plant genera were derived from the European database (Middleton-Welling et al. 2020). The altitudinal range was calculated as the interval between min and max altitude where the species can be found in the Alps (Tolman 2008).

|  | **Altitudinal range (m)** | **Wingspan average (mm)** | **Voltinism** | **Host plant genera (N)** |
| --- | --- | --- | --- | --- |
| **Min** | 0 | 22.50 | Categorical var. | 1.000 |
| **1st Qu.** | 1400 | 30.00 | 3 categories: | 1.000 |
| **Median** | 1750 | 36.00 | Univoltine  Bivoltine  Multivoltine | 3.000 |
| **Mean** | 1726 | 38.54 |  | 4.298 |
| **3st Qu.** | 2100 | 45.00 |  | 5.000 |
| **Max** | 3200 | 77.00 |  | 48.000 |
| **NA** | 0 | 0 | 0 | 6 |

**Table S4.** Results from the GAM1 of records over the years in relation to the sources. Signif. codes: 0 ‘***’ 0.001 ‘**’ 0.01 ‘*’ 0.05 ‘.’ 0.1 ‘ ’ 1.

| **Records** | **Estimate** | **Std. Error** | **z value** | **p-value** |
| --- | --- | --- | --- | --- |
| **Intercept** | 1.459 | 0.299 | 4.876 | <0.001 *** |

| **Records (smooth terms)** | edf | Ref.df | Chi.sq | p-value |
| --- | --- | --- | --- | --- |
| **Citizen Science** | 5.839 | 6.966 | 317.2 | <0.001 *** |
| **Literature & Collections** | 8.85 | 8.991 | 457.5 | <0.001 *** |
| **Sampling** | 5.248 | 6.333 | 305 | <0.001 *** |

**Table S5.** Results from the GAM2 of species richness over the years in relation to the sources. Signif. codes: 0 ‘***’ 0.001 ‘**’ 0.01 ‘*’ 0.05 ‘.’ 0.1 ‘ ’ 1.

| **Species richness** | **Estimate** | **Std. Error** | **z value** | **p-value** |
| --- | --- | --- | --- | --- |
| **Intercept** | 1.459 | 0.299 | 4.876 | <0.001 *** |

| **Species richness (smooth terms)** | edf | Ref.df | Chi.sq | p-value |
| --- | --- | --- | --- | --- |
| **Citizen Science** | 5.066 | 6.122 | 271.9 | <0.001 *** |
| **Literature & Collections** | 8.216 | 8.664 | 376.6 | <0.001 *** |
| **Sampling** | 4.776 | 5.793 | 252.8 | <0.001 *** |

**Ecological trait and source analysis**

**Table S6.** Results from the GLMM of species records per source in relation to functional traits. Signif. codes: 0 ‘***’ 0.001 ‘**’ 0.01 ‘*’ 0.05 ‘.’ 0.1 ‘ ’ 1  **​​**

| **Records** | **Chisq** | **Df** | **p-value** |
| --- | --- | --- | --- |
| **Source** | 113.410 | 2 | <0.001 *** |
| **Voltinism** | 2.856 | 2 | 0.240 |
| **Host plant Genera** | 1.580 | 1 | 0.209 |
| **Altitudinal range** | 5.849 | 1 | 0.016 * |
| **Wingspan** | 0.277 | 1 | 0.599 |
| **Source x Voltinism** | 3.217 | 4 | 0.522 |
| **Source x Host plant Genera** | 1.998 | 2 | 0.368 |
| **Source x Altitudinal range** | 0.696 | 2 | 0.706 |
| **Source x Wingspan** | 4.353 | 2 | 0.114 |

**Table S7.** Post hoc results from the GLMM without non-significant interaction terms of species records in relation to sources. Signif. codes: 0 ‘***’ 0.001 ‘**’ 0.01 ‘*’ 0.05 ‘.’ 0.1 ‘ ’ 1

| **Records** | **Estimate** | **Std. Error** | **z value** | **p-value** |
| --- | --- | --- | --- | --- |
| **Literature - CS** | -0.355 | 0.127 | -2.796 | 0.014 * |
| **Sampling - CS** | -1.268 | 0.125 | -10.174 | <0.001 *** |
| **Sampling - Literature** | -0.912 | 0.133 | -6.872 | <0.001 *** |

**Table S8**. Results from the GLMM of PETS extinction risk divided for source in relation to functional traits. Signif. codes: 0 ‘***’ 0.001 ‘**’ 0.01 ‘*’ 0.05 ‘.’ 0.1 ‘ ’ 1

| **Extinction risk** | **Chisq** | **Df** | **p-value** |
| --- | --- | --- | --- |
| **Source** | 9.918 | 2 | 0.007 ** |
| **Voltinism** | 5.735 | 2 | 0.057 . |
| **Host plant Genera** | 0.131 | 1 | 0.717 |
| **Altitudal range** | 8.124 | 1 | 0.004 ** |
| **Wingspan** | 0.018 | 1 | 0.892 |
| **Source x Voltinism** | 2.961 | 4 | 0.564 |
| **Source x Host plant Genera** | 0.195 | 2 | 0.907 |
| **Source x Altitudinal range** | 0.103 | 2 | 0.950 |
| **Source x Wingspan** | 0.038 | 2 | 0.981 |

**Table S9.** Post hoc results from the GLMM without non-significant interaction terms of species records in relation to sources. Signif. codes: 0 ‘***’ 0.001 ‘**’ 0.01 ‘*’ 0.05 ‘.’ 0.1 ‘ ’ 1

| **Extinction risk** | **Estimate** | **Std. Error** | **z value** | **p-value** |
| --- | --- | --- | --- | --- |
| **Literature - CS** | 0.731 | 0.226 | 3.239 | 0.003 ** |
| **Sampling - CS** | 0.5710 | 0.227 | 2.513 | 0.032 * |
| **Sampling - Literature** | -0.160 | 0.170 | -0.943 | 0.610 |

**
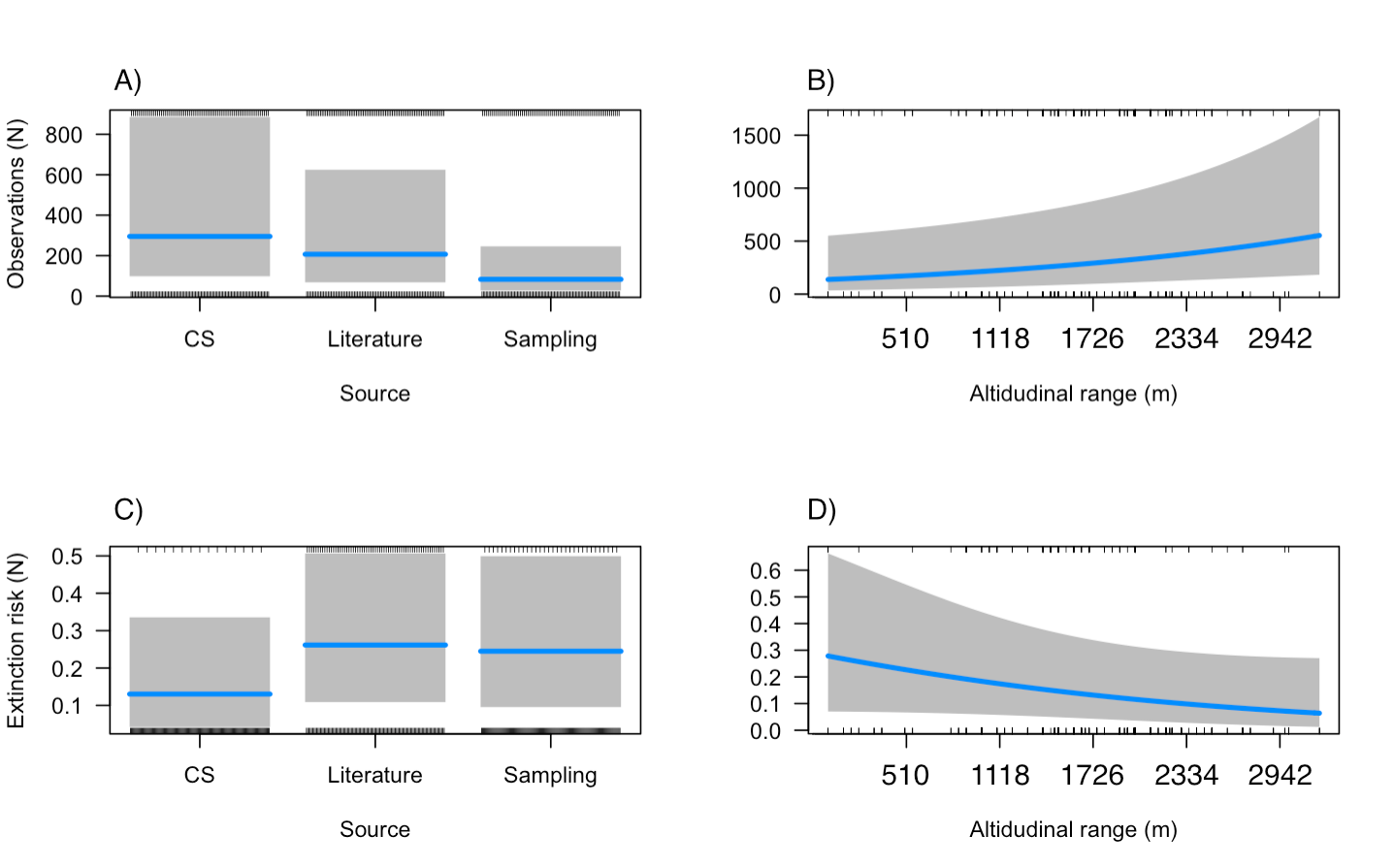
**

**Fig. S5.** Observations changed in relation to source categories (A) and increased with increasing altitudinal range (B). Extinction risk changed in relation to source category (C) and decreased increasing the altitudinal range (D). Lines represent the best-fit models, shadows are the 95% confidence interval. Graphs were drawn using the ‘visreg’ package in R.

**Ecological trait analysis**

**Table S10.** Results from the GLMM of species records in relation to functional traits. Signif. codes: 0 ‘***’ 0.001 ‘**’ 0.01 ‘*’ 0.05 ‘.’ 0.1 ‘ ’ 1

| **Species records** | **Chisq** | **Df** | **p-value** |
| --- | --- | --- | --- |
| **Voltinism** | 5.021 | 2 | 0.081 . |
| **Host plant Genera** | 1.011 | 1 | 0.315 |
| **Altitudinal range** | 3.649 | 1 | 0.056 . |
| **Wingspan** | 2.432 | 1 | 0.119 |

**Table S11.** Results from the GLMM of species extinction risk in relation to functional traits. Signif. codes: 0 ‘***’ 0.001 ‘**’ 0.01 ‘*’ 0.05 ‘.’ 0.1 ‘ ’ 1

| **Extinction risk** | **Chisq** | **Df** | **p-value** |
| --- | --- | --- | --- |
| **Voltinism** | 7.701 | 2 | 0.021 * |
| **Host plant Genera** | 0.099 | 1 | 0.75247 |
| **Altitudinal range** | 3.924 | 1 | 0.048 * |
| **Wingspan** | 0.017 | 1 | 0.896 |

**Table S12.** Post hoc results from the GLMM of species extinction risk in relation to ecological traits. Signif. codes: 0 ‘***’ 0.001 ‘**’ 0.01 ‘*’ 0.05 ‘.’ 0.1 ‘ ’ 1

| **Extinction risk** | **Estimate** | **Std. Error** | **z value** | **p-value** |
| --- | --- | --- | --- | --- |
| **Multivoltine - Bivoltine** | 0.987 | 0.634 | 1.558 | 0.261 |
| **Univoltine - Bivoltine** | -0.582 | 0.492 | -1.185 | 0.459 |
| **Univoltine - Multivoltine** | -1.569 | 0.571 | -2.748 | 0.016 * |

References

Middleton-Welling, J., Dapporto, L., García-Barros, E., Wiemers, M., Nowicki, P., Plazio, E., Bonelli, S., Zaccagno, M., Šašić, M., Liparova, J., & others. (2020). A new comprehensive trait database of European and Maghreb butterflies, Papilionoidea. Scientific Data, 7(1), 351.

Tolman, T. (2008). Collins butterfly guide. HarperCollins UK.
